# Metagenomic Analysis of Virus Diversity and Relative Abundance in a Eutrophic Freshwater Harbour

**DOI:** 10.3390/v11090792

**Published:** 2019-08-28

**Authors:** Christine N. Palermo, Roberta R. Fulthorpe, Rosemary Saati, Steven M. Short

**Affiliations:** 1 Department of Biology, University of Toronto Mississauga, 3359 Mississauga Road, Mississauga, ON L5L 1C6, Canada; 2 Department of Biological Sciences, University of Toronto Scarborough, 1265 Military Trail, Scarborough, ON M1C 1A4, Canada

**Keywords:** viral ecology, freshwater environments, microbial communities, mimiviruses, virophages

## Abstract

Aquatic viruses have been extensively studied over the past decade, yet fundamental aspects of freshwater virus communities remain poorly described. Our goal was to characterize virus communities captured in the >0.22 µm size-fraction seasonally and spatially in a freshwater harbour. Community DNA was extracted from water samples and sequenced on an Illumina HiSeq platform. Assembled contigs were annotated as belonging to the virus groups (i.e., order or family) Caudovirales, Mimiviridae, Phycodnaviridae, and virophages (Lavidaviridae), or to other groups of undefined viruses. Virophages were often the most abundant group, and discrete virophage taxa were remarkably stable across sites and dates despite fluctuations in Mimiviridae community composition. Diverse Mimiviridae contigs were detected in the samples and the two sites contained distinct Mimiviridae communities, suggesting that Mimiviridae are important algal viruses in this system. Caudovirales and Phycodnaviridae were present at low abundances in most samples. Of the 18 environmental parameters tested, only chlorophyll a explained the variation in the data at the order or family level of classification. Overall, our findings provide insight into freshwater virus community assemblages by expanding the documented diversity of freshwater virus communities, highlighting the potential ecological importance of virophages, and revealing distinct communities over small spatial scales.

## 1. Introduction

Viruses can modify and control the structure and function of ecosystems and, in turn, influence global biogeochemical cycles and the evolution of organisms [1]. Historically, the use of traditional culture-based methods to study environmental viruses led to underestimations of their abundance and ecological importance [2], whereas more contemporary molecular methods allowed analyses of environmental microbial communities without many of the constraints of cultivation. However, unlike the prokaryotes and eukaryotes they infect, viruses do not share universally conserved genes that can be readily targeted to survey entire communities. Thus, viral ecology is a field in which major advances can be realized through shotgun metagenomic sequencing [3]. Nonetheless, despite intensive efforts over the past couple of decades, comprehensive knowledge of virus community diversity and dynamics remains elusive for most natural settings.

In the first viral metagenomics study, over 65% of sequences recovered from surface seawater samples were not significantly similar to any sequence in existing databases, highlighting the lack of knowledge of environmental viruses [4]. Since then, viral sequence databases have expanded dramatically, largely due to several large-scale ocean sampling expeditions that included viral community analysis (as reviewed in [5]). These sampling expeditions include the Tara Oceans Expedition, the Malaspina Circumnavigation Expedition, Pacific Ocean Virome (POV), the San Pedro Ocean Time-Series (SPOT), and the Bermuda Atlantic Time-Series Study (BATS). BATS tracked viral abundance in the Sargasso Sea over a decade, while the SPOT studies measured temporal variation in virus communities and their hosts. The POV was established with data from transects spanning from coastal waters to the open ocean to document spatial changes in microbial and virus communities, whereas the Tara Oceans and Malaspina Circumnavigation Expeditions were designed to gather baseline global oceanic biodiversity data.

The Tara Oceans Expedition was conducted from 2009–2013 with the aim of globally sampling a wide range of organismal and functional diversity in the surface oceans, while the Malaspina Circumnavigation Expedition sailed from December 2010 to July 2011 with a focus on deep ocean microbiology. The Tara Oceans Expedition resulted in several important discoveries related to diverse groups of marine planktonic taxa, including viruses (e.g., [6,7,8,9,10]). Importantly, this sampling expedition provided data supporting previous observations of high local diversity but limited global diversity, which led to the conception of a seed-bank model of virus diversity [11]. The Tara Oceans survey revealed that virus community composition was strongly impacted by temperature and oxygen concentrations on local scales due to the influence of these factors on their hosts, while on larger scales ocean currents were responsible for transporting and mixing a virus seed-bank [6]. Furthermore, using 17 viromes generated from the expedition, the abundance and diversity of nucleo-cytoplasmic large DNA viruses (NCLDVs) were mapped, revealing that there were approximately 10^4^–10^5^ viruses per mL in the photic zone and that the so-called “Megavirales” and Phycodnaviridae were the most common NCLDVs in the epipelagic oceans [12]. Complementing the Tara Oceans Expedition, data stemming from the Malaspina Circumnavigation Expedition demonstrated that viruses had higher turnover rates in the deep ocean compared to the surface waters, and they played important roles in dissolved organic carbon (DOC) production and nutrient release, especially in the bathypelagic [13]. By combining viral sequences from the Tara Oceans and the Malaspina Circumnavigation Expeditions, numerous virus genomes have been assembled, primarily from small DNA phages. These efforts expanded viral sequence databases more than three-fold [14], vastly improving our understanding of marine viral ecology and highlighting the global importance of virus activity.

Though extensive surveys of marine virus communities have been conducted, relatively little is known about the fundamental aspects of freshwater virus ecology, such as their distribution in the environment. Despite their underrepresentation in databases, research has demonstrated that freshwater virus communities contain novel viruses and are distinct from other aquatic virus communities [15,16,17]. Metagenomics has been used to study virus communities in natural freshwater lakes from the Arctic [18], Canada [19], the USA [20,21], Ireland [22], France [17], China [23], and Antarctica [24,25]. With respect to virus communities in eutrophic lakes, studies by Green et al. [20], Skvortsov et al. [22], and Ge et al. [23] revealed that virus communities were dominated by Caudovirales in the epilimnion of eutrophic lakes in USA, Ireland, and China, respectively, but other dsDNA viruses, unclassified bacteriophages, and ssDNA viruses were also detected, albeit at lower abundances. Roux et al. [26] mined metagenomic datasets to study virophage and NCLDV communities in a eutrophic freshwater lake in the USA, and observed highly dynamic virophage communities lacking any apparent annual or seasonal patterns of abundance. Although viral sequence databases have expanded rapidly alongside knowledge of marine virus ecology, documenting virus diversity and activity in disparate freshwater environments remains an exciting and untapped area for research.

The goal of the research described here was to characterize the virus community in Hamilton Harbour, a eutrophic freshwater embayment of Lake Ontario. The harbour is located at the western end of Lake Ontario and has an area of 21.5 km^2^ and an average depth of 13 m. It is separated from Lake Ontario by a naturally occurring sandbar and the Burlington Shipping Canal [27] and is the largest Canadian port in the Great Lakes [28]. The area surrounding the harbour has a long history of industrial activity, resulting in a highly polluted harbour containing heavy metals and many other hazardous contaminants [29,30]. Moreover, inputs from wastewater treatment plants, stormwater and sewage overflow, and agricultural and urban runoff have led to high nutrient concentrations, especially phosphorus. Thus, Hamilton Harbour is a eutrophic system that experiences seasonal blooms of cyanobacteria and algae, poor water clarity, and depleted hypolimnetic oxygen. As a result of all these perturbations, the harbour was designated an “Area of Concern” in the amended 1987 USA–Canada Great Lakes Water Quality Agreement (GLWQA). Despite its designation over 30 years ago and ongoing remediation efforts, Hamilton Harbour remains one of the most impaired sites in the Canadian Great Lakes [31].

While Hamilton Harbour is in general an extensively studied system, the microbial community has only been examined using microscopic techniques to investigate microbial diversity and abundance. To our knowledge, there are no published studies characterizing the microbial community in Hamilton Harbour using metagenomics, nor are there any studies of the virus community in Hamilton Harbour. Traditional virome studies have analyzed the <0.22 µm size-fraction, neglecting larger viruses like many of the NCLDVs, as well as viruses contained within cells or associated with particles. The metagenomic dataset used in this study was originally generated to study Hamilton Harbour bacteria and eukaryotic plankton. However, recognizing that this dataset provided a unique opportunity to explore large (>0.22 µm diameter) viruses, as well the as cell- and particle-associated virus community, the data were mined for viral sequences. This allowed us to determine seasonal and spatial patterns of diversity and abundance in this unique freshwater environment. More research is required to better characterize freshwater virus diversity, community structures, and patterns of abundance, and the factors that drive these phenomena. This research aims to address some of these knowledge gaps by providing a detailed view of the >0.22 µm size-fraction virus community in Hamilton Harbour.

## 2. Materials and Methods

### 2.1. Sampling Sites and Collection

Water samples were collected from two long-term Environment Canada monitoring sites in Hamilton Harbour (referred to as stations 1001 and 9031 in previous publications). One site (station 1001) is located at the deepest and most central part of the harbour (43°17′17.0″ N 79°50′23.0″ W) with a water depth of 24 m and a 1.2 km distance from the shoreline. The other site (station 9031) is located less than 0.5 km from the shoreline (43°16′50.0″ N 79°52′32.0″ W), with a water depth of 12 m. This “nearshore” site is influenced by effluents from the Cootes Paradise watershed on the west end of the harbour, while the “mid-harbour” site is closer to the Burlington Shipping Canal and Lake Ontario. In 2015, water samples for metagenomic analyses were collected on 30 July 30, 13 August, 27 August, 10 September, and 24 September from both sites at approximately 1 m below the surface using a Van Dorn bottle sampler; in total, 10 samples were collected, 5 from each of the nearshore and mid-harbour sites. Water samples of 500 mL were filtered through 0.22 μm pore-size Sterivex capsule filters (MilliporeSigma, Burlington, MA, USA), and the filters were sealed and stored at −80 °C until further analysis. For each sample collected, physiochemical parameters including pH, dissolved oxygen, temperature, redox potential, and chlorophyll a were measured in situ using a YSI 58 (Xylem Inc., Rye Brook, NY, USA). Secchi depth was also measured with each sample.

### 2.2. DNA Extraction and Sequencing

Within a month of each sample collection date, samples were removed from storage at −80 °C and allowed to thaw at room temperature prior to DNA extraction from the Sterivex filters. Biomass was recovered from the filters by adding 2 mL of molecular grade nuclease-free water to each filter and vortexing for 5 min. All resuspended material was sequentially transferred to a 1.5 mL microcentrifuge tube under sterile conditions and centrifuged at 6000× *g* for a total of 15 min. DNA was extracted from the pelleted material using a FastDNA SPIN Kit (MP Biomedicals, Solon, OH, USA), beginning with the addition of 500 μL of CLS-Y solution. The manufacturer’s protocol was followed, except the wash step was repeated three times to maximize the removal of environmental contaminants. Following extraction, DNA concentrations were estimated using a NanoDrop ND-1000 UV-Vis Spectrophotometer (NanoDrop Technologies, Inc., Wilmington, DE, USA), and were standardized to 1.5 μg of DNA per sample before being sent for library preparation and shotgun metagenome sequencing by MR DNA (Molecular Research LP, Shallowater, TX, USA).

Sample DNA libraries were prepared in December 2015 using a Nextera DNA Sample Preparation Kit (Illumina, San Diego, CA, USA), following the manufacturer’s protocol. DNA concentrations were measured using the Qubit dsDNA HS Assay Kit (Life Technologies, Carlsbad, CA, USA) before and after library preparation (Table 1). Prior to library preparation, samples were diluted to achieve the recommended concentration of 2.5 ng/μL. For the 27 August nearshore and mid-harbour samples, achieving a concentration of 2.5 ng/μL was not possible, so the maximum volume (20 μL) of each sample was used for these libraries. Average library size was determined using an Agilent 2100 Bioanalyzer (Agilent Technologies, Santa Clara, CA, USA). Each library was clustered using a cBot System (Illumina, San Diego, CA, USA), and was sequenced from paired ends using 500 cycles with the HiSeq 2500 (2 × 250 bp) system (Illumina, San Diego, CA, USA).

### 2.3. Metagenome Data Processing

After sequencing and base-calling, adapters and barcodes were removed by MR DNA (Molecular Research LP, Shallowater, TX, USA). Read quality parameters were verified using FastQC version 0.11.5 [32] prior to quality control and once again prior to assembly. Quality control was performed using a sliding window method with the program Sickle version 1.33 [33] using a quality score cut-off value of 30. All reads shorter than 50 bp were removed prior to assembly. Reads were assembled using IDBA-UD version 1.1.3 [34] with alterations to the source code to accommodate longer read lengths and higher maximum k values (as in [22]). The de Bruijn graph-based assembly was performed using k values from 20 to 200 in increments of 20. A minimum k-mer count of 1 was used in to maximize assembly of the low coverage reads. Alignment of contigs greater than 200 bp was achieved with a BLASTx search against the April 2018 NCBI-nr database downloaded from ftp://ftp.ncbi.nih.gov/blast/db/FASTA/nr.gz. DIAMOND version 0.9.19 [35] was used for alignment with frameshift alignment and very sensitive modes activated. MEGAN6-LR version 6.11.4 [36] was used to annotate contigs using the Lowest Common Ancestor (LCA) algorithm in long read mode with a bit score cut-off value of 100 and a 10^−6^ e-value cut-off. The March 2018 MEGAN protein accession mapping file was downloaded from http://ab.inf.uni-tuebingen.de/data/software/megan6/download/welcome.html. Quality-controlled reads were mapped back to assembled contigs using Bowtie 2 [37] in very sensitive mode, and mapping information for each contig was extracted using SAMtools [38]. Table 2 summarizes the number of reads and contigs at each step in the pipeline for each sample. Mean, standard deviation, and median contig lengths were generated using the application Compute Contig Statistics in the CyVerse Discovery Environment [39]. The DNA sequences generated in this study were submitted to the MG-RAST server under the following accession numbers: mgm4683865.3, mgm4683866.3, mgm4683867.3, mgm4683868.3, mgm4683869.3, mgm4683870.3, mgm4683871.3, mgm4683872.3, mgm4683873.3, mgm4683874.3.

### 2.4. Metagenome Data Analysis

Contig relative abundances were estimated after accounting for differences in contig lengths and sequencing depth (i.e., total number of reads) per sample. More explicitly, the number of reads that mapped to each contig was divided by the contig length. Then, the relative proportion of each contig within a sample was calculated by dividing the normalized number of reads for that contig by the normalized number of reads for all contigs in the sample. All virus contigs were sorted into one of the following categories based on the NCBI taxonomic classifications: Caudovirales, Mimiviridae, Phycodnaviridae, virophages (Lavidaviridae), Iridoviridae, Poxviridae, other dsDNA viruses, ssDNA viruses, unclassified bacterial viruses, and unclassified viruses. Because the assigned taxonomic classifications for some contigs in the “unclassified bacterial viruses” and “unclassified viruses” categories were less specific than information provided in the literature within which they were originally reported, some contigs in these categories were manually curated and assigned to more specific groups based on published information (Table S1).

As well as assigning some contigs to more specific categories, some of the more specific assignments in the dsDNA viruses group were reassigned to the “other dsDNA viruses” group if they were observed in less than half of the samples and at <0.5% abundance. For example, contigs annotated as Marseilleviridae were only observed in 2 of the 10 samples at <0.2% abundance. Because there was not enough representation to making meaningful comparisons, the Marseilleviridae were grouped together with the “other dsDNA viruses”. As a final note, in one sample, a single contig was annotated as an RNA virus and reassigned to the “unclassified viruses” category (Table S1).

There is evidence that some viruses previously and tentatively considered phycodnaviruses (i.e., members of the Phycodnaviridae family) are more closely related to Mimiviridae than Phycodnaviridae, and in fact form a separate subfamily “Mesomimivirinae” within the Mimiviridae family [40,41]. Proposed members of the “Mesomimivirinae” include Organic Lake phycodnaviruses (OLPVs), *Aureococcus anophagefferens* virus (AaV), *Chrysochromulina ericina* virus (CeV), *Phaeocystis pouchetii* virus (PpV), *Pyramimonas orientalis* virus (PoV), and Group I *Phaeocystis globosa* viruses (PgVs) [40,41,42,43,44]. Here, we use the term Mimiviridae to encompass the formally recognized Mimiviridae as well as the proposed “Mesomimivirinae” subfamily that are often considered to be phycodnaviruses. Based on these recent developments, we manually curated the affiliation of viruses that belong to the proposed Mesomimivirinae group but were annotated as Phycodnaviridae in the NCBI scheme (accessed April 2018) as viruses within the Mimiviridae family (Table S1).

A boxplot of the overall virus community was generated using the “ggplot2” package [45] in RStudio. Bray–Curtis dissimilarity with the unweighted pair group method with arithmetic mean (UPGMA) clustering was used to determine which samples were most similar based on relative abundances of different virus groups. The relationship between the relative abundance of virus groups, environmental parameters (dissolved oxygen, pH, chlorophyll a, temperature, Secchi depth, and redox potential), and sites was statistically tested using canonical correspondence analysis (CCA); tests of 10,000 permutations were used to compute the significance of the model and the variables. Cluster analysis and CCA were computed using the “vegan” package [46] in RStudio. For samples collected on 30 July, 10 September, and 24 September, data were available for other factors, including ammonia, chloride, fluoride, sulfate, particulate organic carbon, particulate organic nitrogen, nitrate and nitrite, total dissolved nitrogen, total phosphorus, total dissolved phosphorus, total particulate phosphorus, and soluble reactive phosphorus. Separate CCA models were tested for the entire dataset and the subset of dates for which additional data were available.

## 3. Results

### 3.1. Virus Community Composition in Hamilton Harbour

**Figure 1:**
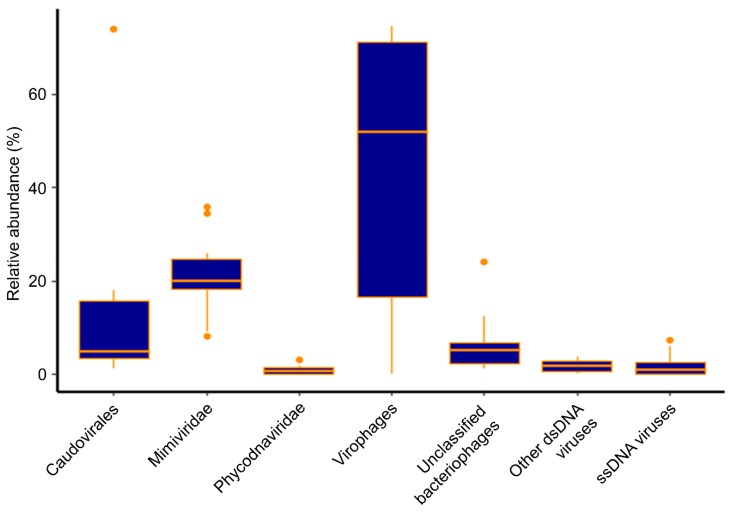
Boxplot of the overall virus community in Hamilton Harbour based on all 10 metagenomes from the 5 sampling dates at the nearshore and mid-harbour sites.

**Figure 2:**
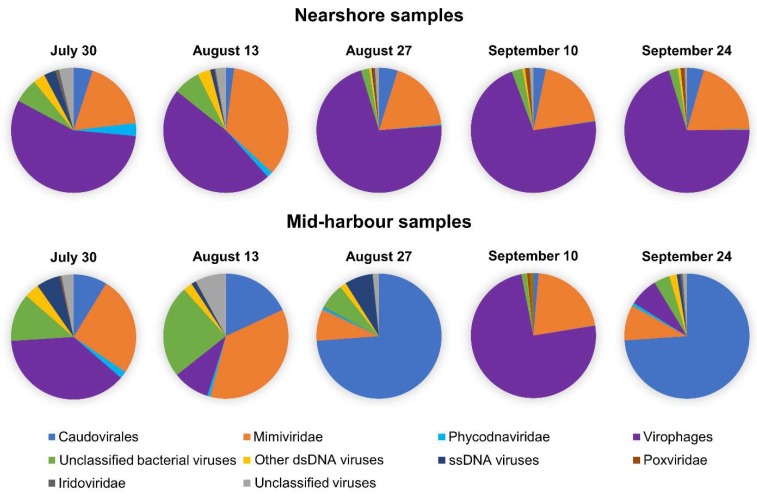
Individual virus communities for each of the 10 metagenomes.

Diverse virus contigs were identified in Hamilton Harbour and some were classified within the virus groups Caudovirales, Mimiviridae, Phycodnaviridae, and virophages (Lavidaviridae), while others could only be classified as belonging to “unclassified bacteriophages”, “other dsDNA viruses”, and “ssDNA viruses”. Henceforth, for the sake of brevity, we name virus groups only when referring to contigs annotated as viruses from those groups. Overall, virophages were the most abundant (44.7% average abundance across all samples), but were also the most variable (from 0.1% to 74.5% abundance). The Mimiviridae were the second most abundant group, comprising an average of 21.1% of the virus community across all samples. Interestingly, though they were intimately associated, the abundances of Mimiviridae (ranging from 8.2% to 36.0% abundance) did not fluctuate to the same extent as the virophages (Figure 1). At the nearshore site, virophages represented a large percentage of the virus community in every sample, with abundances between 47.4% and 71.7%, yet their abundances fluctuated widely at the mid-harbour site, ranging from 0.12% to 74.5%. Mimiviridae were consistently detected as a substantial proportion of the community in the nearshore samples, with relative abundances ranging from 18.2% to 34.6% overall. Again, like the virophages, Mimiviridae abundances were more variable at the mid-harbour site, comprising between 8.2% and 36.0% of all virus contigs. Similarly, Caudovirales consistently represented less than 5.0% of the virus community in the nearshore samples, but ranged from 1.3% to 73.9% at the mid-harbour site. Phycodnaviridae were a minor component of the virus community in all samples, representing only 0.09% to 3.3% at the nearshore site and 0.05% to 1.9% at the mid-harbour site (Figure 2).

**Figure 3:**
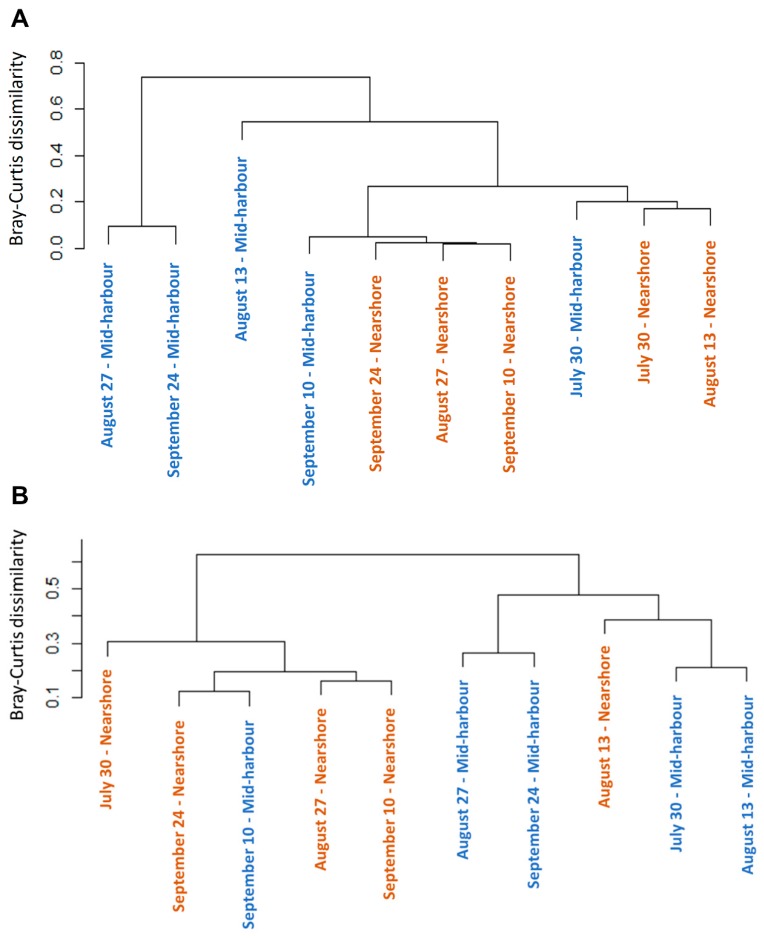
Dendrogram of cluster analysis based on Bray–Curtis dissimilarity index of the (**A**) overall virus community and (**B**) Mimiviridae community. Nearshore samples are coloured orange and mid-harbour samples are coloured blue.

In general, the abundance of different groups of viruses was more variable in the mid-harbour compared to the nearshore samples. Sample similarity based on community composition was assessed using UPGMA clustering of a Bray–Curtis dissimilarity matrix, which reinforced the contrast of the nearshore and mid-harbour sites (Figure 3A). Though the nearshore and mid-harbour samples did not form distinct clusters overall, the nearshore samples clustered more closely together than the mid-harbour samples. The community composition at the two sites clustered together on 30 July and 10 September, but resolved to distinct clusters on 13 August, 27 August, and 24 September.

### 3.2. Mimiviridae Community Composition in Hamilton Harbour

**Figure 4:**
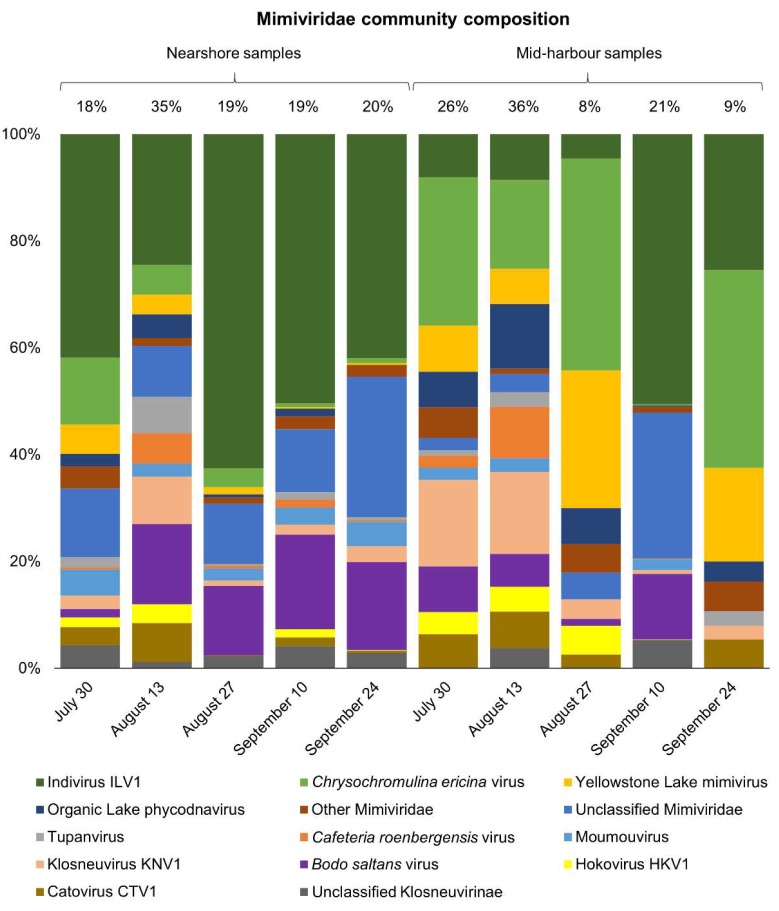
Mimiviridae community composition at the nearshore and mid-harbour sites from 30 July to 24 September. Numbers above bars are percentages of the total virus community that the entire bar represents.

Diverse Mimiviridae contigs were detected in Hamilton Harbour, including representatives of all subgroups and proposed subgroups. Most notably, the proposed “Klosneuvirinae” subfamily [47] comprised a large proportion of the Mimiviridae community, ranging from 17.5% to 79.0% across all samples, and representing an average of 67.4% and 41.7% of the Mimiviridae community at the nearshore site and mid-harbour site, respectively. In general, the nearshore and mid-harbour sites appeared to host distinct Mimiviridae communities, a notion supported the Bray–Curtis dissimilarity clustering analysis (Figure 3B); samples from the two sites clustered separately, with the exceptions of 10 September at the mid-harbour site and 13 August at the nearshore site. In all nearshore samples and on 10 September at the mid-harbour site, Indivirus ILV1 was the most abundant representative of the Mimiviridae community, while *Chrysochromulina ericinia* viruses (CeV) were the most abundant Mimiviridae in all mid-harbour samples, except on 10 September (Figure 4) when *Indivirus ILV1* was again dominant.

Of the proposed subfamily “Mesomimivirinae”, Organic Lake phycodnavirus, Yellowstone Lake mimivirus, and *Chrysochromulina ericinia* virus were the most abundant. The “other Mimiviridae” category was created for Mimiviridae that were present in only a few of the samples and always at less than 2.0% of the community, and included the following Mesomimivirinae members: *Aureococcus anophagefferens*, *Phaeocystis pouchetii*, *Pyramimonas orientalis*, and *Phaeocystis globosa*. Also placed in the “other Mimiviridae” category were *Acanthamoeba polyphaga* mimivirus, unclassified Megaviridae, Megavirus Iba, Powai Lake megavirus, and Mimivirus AB-566-O17, which were detected in six of the samples at less than 6.0% relative abundance.

### 3.3. Virophage Community Composition in Hamilton Harbour

**Figure 5:**
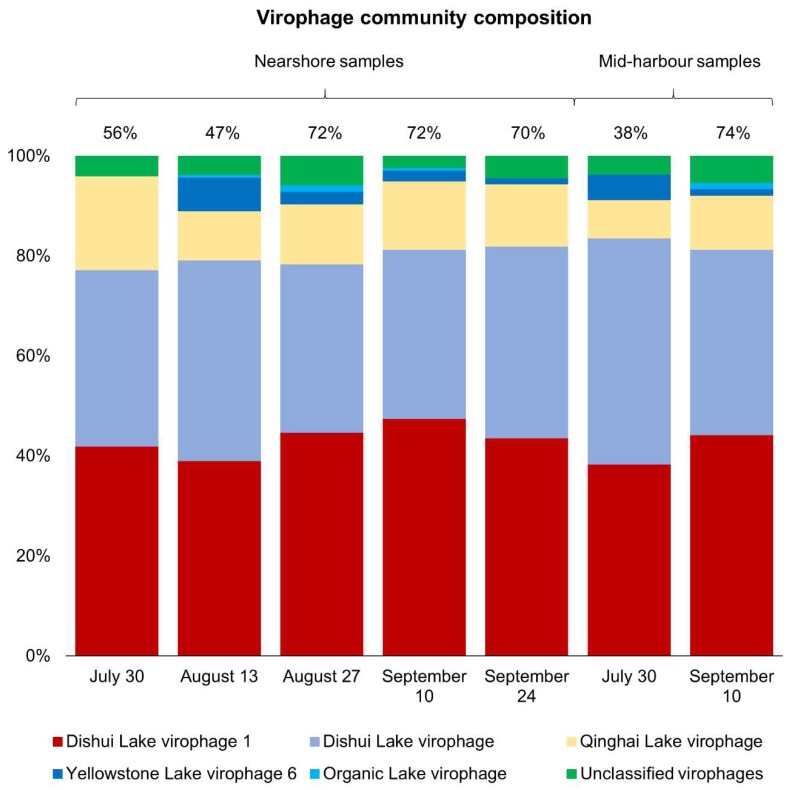
Virophage community composition in samples where virophages represent >10% of the total virus community. Numbers above bars are percentages of the total virus community that the entire bar represents.

Given the similarity of virus community composition in the nearshore samples, we explored whether this consistency was upheld at the level of discrete taxa in the most abundant group, the virophages. Across all nearshore samples, virophage community composition was very similar regardless of date and contigs were annotated as five types of virophages: Dishui Lake virophage, Dishui Lake virophage 1, Yellowstone Lake virophage 6, Qinghai Lake virophage, and Organic Lake virophage (Figure 5). Across all samples, the majority of virophage contigs were most similar to Dishui Lake virophage 1 (43.4% average) and Dishui Lake virophage (36.3% average). Qinghai Lake virophages comprised 13.3% of the communities on average, and the Yellowstone Lake virophages were observed at lower abundances averaging only 2.5% of the virophage community. Least abundant were the Organic Lake virophages, which were detected at 0.5% abundance on average and were only detectable in three of the five nearshore samples. Unclassified virophage contigs were also detected at low abundances on all dates, averaging 4.0% of the virophage community. Overall, from 30 July to 24 September, the virophage community composition at the nearshore site was remarkably similar. In contrast to virophage communities at nearshore sites, the mid-harbour samples from 30 July and 10 September were the only samples with virophage populations comprising >10% of the total virus community. Again, both samples were dominated by the Dishui Lake virophages (Dishui Lake virophage 1 and Dishui Lake virophage), which comprised about 80% of the total virophage population. Regardless of site or date, the contigs most closely resembled virophages originally identified in Dishui Lake, China (Figure 5).

### 3.4. Influence of Environmental Parameters on Virus Community Composition

A canonical correspondence analysis (CCA) was performed to assess the influence of pH, temperature, redox potential, dissolved oxygen, Secchi depth, and chlorophyll a on the viral groups at each site. The CCA model explained 47.8% (F = 7.12, Pr(>F) = 0.005) of the variability in the data. A test of 10,000 permutations revealed that the chlorophyll a concentration was the only significant environmental parameter of those assessed. Temperature, pH, dissolved oxygen, Secchi depth, and redox potential were not significant explanatory factors of changes in virus community composition and relative abundances between samples. However, there was a strong inverse relationship of Caudovirales and chlorophyll a concentration. The 27 August and 24 September mid-harbour samples, which were dominated by Caudovirales (>70%), were similar in community composition (Figure 3A) and were negatively correlated with chlorophyll a. All nearshore samples clustered closely together and were positively correlated with chlorophyll a. Several parameters including ammonia, chloride, fluoride, sulfate, particulate organic carbon, particulate organic nitrogen, nitrate and nitrite, total dissolved nitrogen, total phosphorus, total dissolved phosphorus, total particulate phosphorus, and soluble reactive phosphorus were measured only on 30 July, 10 September, and 24 September at both sites; however, none were significant explanatory variables of the virus communities on these dates. Interestingly, when the influence of environmental variables on the Mimiviridae community was assessed, chlorophyll a remained an explanatory variable, accounting for 37.8% of the variation in the Mimiviridae community (F = 4.85, Pr(>F) = 0.018). Of the parameters measured only on 30 July, 10 September, and 24 September, only particulate organic carbon (POC) significantly explained differences in the Mimiviridae communities between samples. In a separate CCA model, a test of 719 permutations revealed that POC accounted for 59.6% of the variation in the Mimiviridae community on 30 July, 10 September, and 24 September (F = 5.90, Pr(>F) = 0.043).

## 4. Discussion

### 4.1. Influence of Databases

In this study, we observed diverse virus communities that were both spatially and seasonally variable, and often dominated by virophages. However, as usual for environmental metagenomes, and particularly for viruses, a large portion of sequences remained unclassified and were discarded at the annotation step of the data processing pipeline. Viruses have much fewer sequenced representatives in databases than prokaryotes or eukaryotes and are more likely to remain unannotated. For example, in May 2018, RefSeq released 88 contained >7500 viruses with >325,300 associated accessions (9648 nucleotide sequences and 315,742 protein sequences). In contrast, there were >50,400 bacteria with >100,583,400 associated accessions (12,367,951 nucleotide sequences and 88,193,695 protein sequences) (data from: ftp://ftp.ncbi.nlm.nih.gov/refseq/release/release-statistics/). An estimated <1% of the Earth’s virome has been discovered [48], and this underrepresentation in databases has a large influence on the reported diversity of viruses in metagenomes. The NCBI-nr database referenced in this study combines data from RefSeq as well as SwissProt, Protein Information Resource (PIR), Protein Databank (PDB), Protein Research Foundation (PRF), and GenPept. A combination of curated and non-curated sequences, the NCBI-nr database includes data from the assembly of environmental metagenomes, including those deposited by private institutions. The use of this database as opposed to a curated database drastically increases the size of the reference database, therefore increasing the likelihood of annotation, which is especially valuable for identifying taxa with few sequenced representatives. Within viral sequence databases, certain groups of viruses have many sequenced representatives, while others are represented by only a few sequences. For example, there are >2000 complete Caudovirales genomes, but only seven complete virophage (Lavidaviridae) genomes (data from https://www.ncbi.nlm.nih.gov/genomes/GenomesGroup.cgi; retrieved December 2018). This bias could lead to a higher chance of detecting Caudovirales than virophages, which are more likely to be unannotated and discarded in downstream analyses. In other words, it is likely that many virophages exist that have not yet been discovered and these will be missed in our analyses. On the other hand, annotation is also dependent on diversity within a virus group, as well as contig size. Contig size is directly influenced by genome size, hence, viruses with smaller genomes are more likely to generate longer contigs that are more likely to be annotated. In any case, the remarkably high abundance and dynamic nature of virophages detected in Hamilton Harbour suggests their important ecological role in this system.

### 4.2. Limitations of Inferring Virus Presence and Abundance from Metagenomic Datasets

This research infers the presence and abundance of viruses based on the relative abundances of assembled contigs from shotgun sequencing. It is important to note that the presence of contigs annotated as viruses may not necessarily indicate their presence. The intimate evolutionary relationship between viruses and their hosts has resulted in an abundance of viral genes present in host genomes. Unless viral homologues in host genomes are specifically annotated as such, genetic similarity searches may incorrectly annotate them as viruses [49]. However, upon examining the top 10 hits for individual contigs, there were few instances that contained a combination of bacteria and viruses; in most cases the top hits were all within the same virus family.

Metagenomic research is limited by the inability to distinguish between actively infecting virions within cells, inactive virions present freely in the water column or adsorbed to cells or particles, and viral genomes integrated into cellular genomes. Since DNA was extracted from the >0.22 µm fraction rather than the traditional <0.22 µm fraction, it is less likely that the virus contigs captured were derived from free virions in the water column. Most free virions, with the exception of some of the largest NCLDVs, would have passed through the filters, and only those that were within or adsorbed to cells or other particulate matter would have been captured during sample collection. Particularly in environments containing high suspended solids such as Hamilton Harbour, particle adsorption may be an important virus transport mechanism [50]. Indeed, there is extensive literature demonstrating that viruses adsorb to a variety of organic and inorganic suspended solids in aquatic environments (reviewed in [51]). Though virophages are small and could pass through the filters if present as free particles in the water column, it is also possible that several virophages could be adsorbed to individual giant virus hosts. For example, the virophage *Sputnik* is frequently observed to be situated within the fibrils of its Mamavirus host, and is speculated to use these fibrils in order to gain entry into the viral host [52]. Additionally, some virophages integrate into the genomes of their eukaryotic hosts, and a single host can contain several copies of an integrated virophage genome [53,54]. More research is required to assess the influence of virophage-host associations on the interpretation of metagenomic data gathered in studies such as this. Regardless, the highly abundant and dynamic virophage community is indicative of their potential influence on the Mimiviridae and eukaryotic host communities in Hamilton Harbour, and likely many other freshwater environments as well.

Though these observations contrast with metagenomic studies in other freshwater lakes, it remains challenging to compare virus communities between studies due to differences in sampling, sequencing, and data processing. Even the most widely-used analysis tools for metagenomic data yield different results [55], highlighting the need for standardized analysis tools and pipelines in order to facilitate ecologically relevant comparisons between studies. Notably, viral metagenomic studies typically extract and sequence community DNA from the <0.22 µm size-fraction, capturing only free virions and missing some of the largest NCLDVs. These studies could not detect the larger free NCLDVs or virions within and adsorbed to cells and particles that are present in the >0.22 µm size-fraction. Our research aimed to capture this often neglected fraction of the viral community. Therefore, our data is likely enriched with sequences from some Mimiviridae and their associated virophages, resulting in different community structures than typical virome studies.

### 4.3. Physiochemical Factors

Environmental variables that typically explain variation in virus communities on a local scale, such as dissolved oxygen and temperature [6], were not significant explanatory variables of our data. This may be a reflection of virus dependence on host populations and the wide host ranges of virus families. For example, Mimiviridae infect hosts ranging from heterotrophic protists like amoeba to photosynthetic protists, or algae. Because key resources for these eukaryotic microorganisms differ (DOC versus light), it might be anticipated that they respond differently to certain environmental factors. Nevertheless, the CCA model with chlorophyll a as a constrained variable explained 47.8% of the variation in the data, while other environmental parameters were non-significant. Chlorophyll a was inversely related to Caudovirales relative abundance, suggesting that most Caudovirales contigs were derived primarily from phages of heterotrophic bacteria rather than cyanobacteria. For example, the mid-harbour samples from 27 August and 24 September had undetectable or very low (0.1 µg/L) chlorophyll a concentrations, respectively, and the virus communities in these samples were dominated by Caudovirales and clustered closely together in both the CCA and the Bray–Curtis dissimilarity analyses. In contrast, the lowest chlorophyll a concentration noted for a nearshore sample was 2.7 µg/L, and the virus community in this sample (13 August) was dominated by virophages and Mimiviridae. It is notable that the nearshore site is closer in proximity to wastewater effluents from Spencer Creek than the mid-harbour site. Although improvements have been made to the wastewater treatment plants surrounding Hamilton Harbour, effluents still contain phosphorus levels above targets set in the Hamilton Harbour Remedial Action Plan (HHRAP) [56]. These nutrient and organic carbon inputs might stimulate microbial growth at the nearshore site, in turn influencing virus communities.

The CCA of different Mimiviridae taxa against environmental parameters revealed that chlorophyll a remained a significant explanatory variable of the Mimiviridae community, reflecting their wide host range, which includes both photosynthetic and non-photosynthetic hosts. For example, the algal virus CeV made up a large portion of the Mimiviridae community at the mid-harbour site, while Indivirus ILV1, a member of the proposed subfamily “Klosneuvirinae”, that were themselves assembled from metagenome sequences, was abundant in nearshore samples and is a suspected protist virus [47]. POC explained almost 60% of the variation in the Mimiviridae communities on 30 July, 10 September, and 24 September. Since POC data were only available for six of the ten samples, it is unclear whether POC would remain a significant explanatory variable if the model included data from all samples.

Hamilton Harbour is known to be a highly variable system [57] that experiences regular seiches [58] and exchange flows with Lake Ontario, especially during the summer [27]. Circulation and mixing in the harbour are primarily controlled by prevailing winds [59]. Modelling of summer circulation patterns in Hamilton Harbour demonstrated the occurrence of a large eddy in the middle of the harbour at the location of our mid-harbour site, while the nearshore site was situated on the perimeter of a smaller eddy located at the western end of the bay, near Cootes Paradise [60]. The location of the sampling sites with respect to these eddies and Hamilton Harbour circulation may contribute to the differences in virus community composition observed at different sites on the same day. However, the circulation patterns in Hamilton Harbour were not known during our sampling period, so the influence of hydrodynamics is purely speculative, but worth considering. Hamilton Harbour is a major Canadian shipping port and large ships entering the harbour from Lake Ontario likely pass through the mid-harbour site on route to the port on the southern shore. Shipping traffic is one of the many factors potentially affecting virus community variability on the sampling dates that could not be considered in the present study. Given the high variability of Hamilton Harbour, it is perhaps unsurprising that the virus communities varied widely between sampling sites and dates. More unexpected was the relatively stable community throughout the mid-summer to late fall at the nearshore site, when bacterial and phytoplankton populations have been observed to be highly dynamic [61,62,63].

### 4.4. The Virophages

Virophages are small dsDNA viruses that co-infect eukaryotic hosts with giant dsDNA viruses [64]. This co-infection has been shown to reduce giant virus fitness, thereby increasing survival of the cellular host [65,66]. Virophages were discovered only a decade ago [66], and little is known about their ecology. There is evidence to suggest that virophage-induced reduction of algal host mortality leads to longer and more frequent algal blooms [67], highlighting the ecological importance of virophages and their potential relevance to our eutrophic study site.

Virophage abundance in freshwater eutrophic lakes varies widely and has been reported as highly abundant in some environments [26], yet only detected at low abundances (<0.05%) [20] or even undetectable in other lakes [22]. Virophage abundances and distributions in lakes do not have clear seasonal or annual patterns of abundance, nor are there established relationships between dominant types of virophages in different environments [26]. In contrast to previous studies of viruses in eutrophic freshwater lakes, virophages were dominant in most of the samples from Hamilton Harbour. This discrepancy is likely due to the different size-fractions analyzed, as previous freshwater studies sequenced the traditional <0.22 µm size-fraction, while our study analyzed the >0.22 µm size-fraction. Though free virophages present in the water column would have passed through the filters, the approach used for the current study had the potential to enrich for virophages associated with eukaryotic and Mimiviridae hosts. About 80% of the virophage community was annotated as Dishui Lake virophages in seven of ten samples, regardless of date or site. Dishui Lake, China, is a eutrophic freshwater lake that is more similar to Hamilton Harbour than the lakes where other types of virophages were observed, such as Organic Lake, an Antarctic hypersaline lake, Qinghai Lake, a saline endorheic basin, or Yellowstone Lake, which receives geothermal inputs. Due in part to their relatively recent discovery, sequence databases are limited with respect to the amount of information available for virophages, hence, specific virophage annotations in our samples may not be accurate. It is likely that the large portion of the virophage community annotated as Dishui Lake virophages are in fact a diverse array of virophages. The breakdown into discrete taxa did, however, reveal which virophages in reference databases were most similar to those detected in our dataset.

Other considerations that may impact the accuracy of virophage annotations include the presence of polintons/polintoviruses and polinton-like viruses (reviewed in [68]). Related to virophages, polintons/polintoviruses are self-synthesizing transposons that have genes that are homologous to virophages and giant viruses and are frequently integrated into eukaryotic genomes. Polinton-like viruses (PLV) are related to polintons and are commonly integrated into the genomes of green algae [68]. Considering their evolutionary relatedness and the presence of homologous genes in conjunction with the limitations of available databases, it would not be surprising to find that some of the virophages detected in our samples may in fact be polintons/polintoviruses or PLVs. Further exploration and sequencing of virophage, polinton, and PLV diversity is required to expand databases and improve accuracy of identification of these entities in metagenomic datasets. As a final comment on virophages, recent genome sequencing of a giant virus isolated from Lake Ontario revealed the presence of three putative virophages [69]. This giant virus and its associated virophages infect the freshwater haptophyte *Chrysochromulina parva*; the *C. parva* virus is a close relative to the Hamilton Harbour mimivirus contigs annotated as *Chrysochromulina ericinia* virus. This observation supports the notion that the contigs assembled from Hamilton Harbour represent bona fide mimivirus-parasitizing virophages.

### 4.5. Ecological Relevance

The importance of viruses in aquatic environments is well documented. These viruses are estimated to kill approximately 10% of the phytoplankton population and up to 50% of the bacterial population in surface marine waters, with greater impacts in high nutrient environments (reviewed in [1,70,71,72,73]). Particularly in eutrophic aquatic systems, viruses are more active and are hypothesized to control host abundance, respiration, and production [74]. They drive host community succession by targeting and lysing abundant members of the community, allowing less competitive species to thrive [75], and influencing fluctuations in dissolved and particulate organic matter pools. Viruses also act as key gene transfer agents that permit host adaptation and drive the evolution of microbial communities [76].

While marine viruses have been studied extensively over the past decade, freshwater viruses have received relatively little attention. Fundamental aspects of freshwater virus ecology, such as their distribution and patchiness in freshwater environments, remain unknown. To our knowledge, this research is the first report of virus communities in Hamilton Harbour, and one of the few studies of virus communities in the Great Lakes. On some dates (e.g., 24 September) virus community composition was very different over the 3 km distance between the sites, while on other dates (e.g., 10 September), the community composition was very similar. Relative abundances of virus orders and families fluctuated at the mid-harbour site much more than the nearshore site, highlighting the high diversity of viruses on local scales and the impact of small-scale environmental differences.

Since the recent discoveries of the Mimiviridae and their virophages, few studies have looked at the relative abundances of these groups over the duration of a season. We captured fluctuations in the Mimiviridae community over the summer and observed vastly different communities on the same date at different locations in the harbour. While the nearshore and mid-harbour samples generally contained distinct Mimiviridae communities, the virophage community composition remained consistent. This highlights the complexity of these ecological relationships and the gaps in our understanding of how these intimately associated viruses interact. The stability of virophage community composition, despite the fluctuating Mimiviridae community, appears to support the hypothesis that virophages and their Mimiviridae hosts are not connected solely by infection. The recent discovery that some virophages frequently enter eukaryotic host cells independently, remaining latent until infection by a giant virus where they then compete with the giant virus for its replication machinery [53], is a possible explanation of the wide range of abundances in the virophage community, while the Mimiviridae community remained relatively stable.

Hamilton Harbour is known to support high algal biomass in the summer and early autumn months. Given the large differences in community composition between the nearshore and mid-harbour sites, Mimiviridae appear to be important and dynamic algal viruses in Hamilton Harbour. The most common algae-infecting Mimiviridae was CeV, which was especially abundant at the mid-harbour site. Interestingly, while our sampling of the >0.22 µm fraction may have enriched for some Mimiviridae, we would not expect this to be the case for CeV since their capsid sizes are <0.22 µm. The “other dsDNA viruses” category did not contribute more than 4% in any individual sample and included mostly Mimiviridae. The small percentage of ssDNA viruses that were detected represented only those that were replicating in cells in the dsDNA form, since only dsDNA was targeted in the library preparation and sequencing. Therefore, the ssDNA viruses may be underrepresented compared to other virus groups which were detected as virions adsorbed to and within particles and cells, in addition to viruses actively replicating within cells.

## 5. Conclusions

Overall, Hamilton Harbour metagenomes included a diverse array of viruses ranging from large dsDNA Mimiviridae to small ssDNA viruses. Relative abundances of virus groups varied widely over relatively small spatial scales within the harbour, with higher consistency in the nearshore samples compared to the mid-harbour samples. Virophage relative abundances ranged widely across all samples, but were the most abundant virus family in most samples. A wide diversity of Mimiviridae was detected in the samples and the two sites appeared to host distinct Mimiviridae communities, suggesting their importance as algal viruses in Hamilton Harbour and likely other freshwater environments. The abundances of discrete virophage taxa were remarkably stable despite the dissimilar Mimiviridae communities at the two sites, highlighting our limited understanding of how these intimately associated viruses interact. Caudovirales and Phycodnaviridae relative abundances were low in most samples, in contrast to other studies of eutrophic freshwater environments. It is important to note the contrasting approach used in this study compared to other viromic studies. We analyzed the >0.22 µm fraction to capture large free virions, virions associated with cells and particulates, and viral genomes integrated into host genomes. Therefore, it is not surprising that the patterns of diversity we observed differed from other viromic studies. Nonetheless, these findings provide additional insights into virus community structures in freshwater environments, expanding the documented diversity of freshwater virus communities, highlighting the potential ecological importance of virophages, and revealing distinct communities over small spatial scales.

## Figures and Tables

**Table 1:** Initial DNA concentration, final library concentration, and average fragment size in the library for each of the 10 samples in this study.

Sample	Initial DNA Concentration (ng/μL)	Library Concentration (ng/μL)	Average Library Size (bp)
30 July—nearshore	5.30	16.2	985
30 July—mid-harbour	2.84	18.3	1070
13 August—nearshore	2.76	17.5	1070
13 August—mid-harbour	3.40	14.5	1000
27 August—nearshore	2.28 *	15.0	950
27 August—mid-harbour	1.42 *	8.94	615
10 September—nearshore	9.58	12.0	1000
10 September—mid-harbour	10.6	14.1	1060
24 September—nearshore	9.10	15.7	1030
24 September—mid-harbour	2.68	17.7	900

* denotes that samples could not be adjusted to 2.5 ng/μL prior to library preparation.

**Table 2:** Summary of the number of reads and contigs at each step in the data processing pipeline.

Sample	Reads Pre-QC	Reads Post-QC	Contigs Post-Assembly (Mean Length; Standard Deviation; Median Length)	Contigs Assigned	Virus Contigs Assigned (Mean Length ± Standard Deviation; Median Length)	Percent of Virus Contigs
30 July—nearshore	13,403,832	11,608,892	480,233 (692; 589; 526)	212,156	349 (681; 326; 563)	0.16
30 July—mid-harbour	13,206,316	11,246,470	433,927 (823; 1540; 563)	256,277	397 (626; 341; 526)	0.15
13 August—nearshore	12,758,364	11,040,814	398,774 (741; 1493; 531)	196,128	488 (767; 405; 650)	0.25
13 August—mid-harbour	13,104,992	11,414,576	416,468 (820; 1060; 571)	273,693	662 (688; 357; 570)	0.24
27 August—nearshore	15,685,750	13,953,600	374,464 (661; 923; 498)	128,233	484 (693; 300; 592)	0.38
27 August—mid-harbour	16,101,196	14,751,904	233,868 (833; 1567; 568)	183,817	232 (731; 460; 555)	0.13
10 September—nearshore	16,228,148	14,257,158	418,490 (609; 848; 487)	110,567	463 (736; 327; 642)	0.42
10 September—mid-harbour	15,411,142	13,561,346	443,692 (568; 765; 474)	64,142	604 (753; 320; 673)	0.94
24 September—nearshore	14,689,040	12,764,018	367,778 (629; 873; 491)	112,144	372 (810; 612; 676)	0.33
24 September—mid-harbour	13,151,390	11,228,970	296,813 (776; 1094; 550)	185,913	104 (870; 1257; 489)	0.06

## Supplementary Materials

The following are available online at https://www.mdpi.com/1999-4915/11/9/792/s1, Table S1: List of original taxonomic annotations and reassigned taxonomic affiliations of virus contigs in Hamilton Harbour metagenomes.
