# Supplementary material for: Metagenomic Analysis of Virus Diversity and Relative Abundance in a Eutrophic Freshwater Harbour
Source: Viruses. 2019 Aug 28;11(9):792. doi: 10.3390/v11090792 (PMC6784016; doi:10.3390/v11090792)
Supplement: Supplementary file 1 [file viruses-11-00792-s001.zip › HH Manuscript 07 04 2019_supplementary materials.pdf]

SUPPLEMENTARY MATERIALS

Table S.1: List of originally assigned taxonomic annotation and re-assigned taxonomic grouping of virus contigs in Hamilton Harbour metagenomes

**Original Annotation**	**Re-assigned Grouping**
root;**Viruses;**	Unclassified viruses
root;Viruses;**ssRNA viruses…**	Unclassified viruses
Unclassified viruses	
root;Viruses;unclassified viruses;**Pacmanvirus A23;**	Other dsDNA viruses
root;Viruses;unclassified viruses;**Faustovirus;**	Other dsDNA viruses
root;Viruses;unclassified viruses;**Sewage-associated circular DNA virus-1;**	Unclassified ssDNA viruses
root;Viruses;unclassified viruses;**Skeletonema virus LDF-2015a;**	Unclassified ssDNA viruses
root;Viruses;unclassified viruses;**Lake Sarah-associated circular virus-1;**	Unclassified ssDNA viruses
dsDNA viruses, no RNA stage	
root;Viruses;**dsDNA viruses, no RNA stage;**	Other dsDNA viruses
root;Viruses;dsDNA viruses, no RNA stage;**Iridoviridae;**	Iridoviridae
root;Viruses;dsDNA viruses, no RNA stage;**Poxviridae;**	Poxviridae
root;Viruses;dsDNA viruses, no RNA stage;**Marseilleviridae;**	Other dsDNA viruses

root;Viruses;dsDNA viruses, no RNA stage;**Tectiviridae;**	Other dsDNA viruses
environmental samples <viruses>	
root;Viruses;**environmental samples <viruses>;**	Unclassified viruses
root;Viruses;environmental samples <viruses>;**Organic Lake virophage;**	Virophages
root;Viruses;environmental samples <viruses>;uncultured environmental isolates;**uncultured Mediterranean phage uvDeep1…**	Unclassified bacteriophages
root;Viruses;environmental samples <viruses>;**uncultured marine virus;**	Unclassified viruses
root;Viruses;environmental samples <viruses>;**uncultured Mediterranean phage uvMED;**	Unclassified bacteriophages
root;Viruses;environmental samples <viruses>;**uncultured Mediterranean phage;**	Unclassified bacteriophages
root;Viruses;environmental samples <viruses>;**uncultured virus;**	Unclassified viruses
root;Viruses;environmental samples <viruses>;uncultured environmental isolates;**uncultured phage MedDCM-OCT-S04-C348;**	Unclassified bacteriophages
root;Viruses;unclassified bacterial viruses;environmental samples <bacteriophages>;**environmental Halophage eHP-25;**	Unclassified bacteriophages
unclassified bacterial viruses	
root;Viruses;**unclassified bacterial viruses;**	Unclassified bacteriophages
root;Viruses;unclassified bacterial viruses;**Agrobacterium phage Atu_ph07;**	Unclassified bacteriophages
root;Viruses;unclassified bacterial viruses;**Bradyrhizobium phage BDU-MI-1;**	Unclassified bacteriophages

root;Viruses;unclassified bacterial viruses;environmental samples <bacteriophages>;**Lake Baikal phage Baikal-20-5m-C28;**	Unclassified bacteriophages
root;Viruses;unclassified bacterial viruses;**Freshwater phage uvFW…**	Unclassified bacteriophages
root;Viruses;unclassified bacterial viruses;**Gordonia phage GMA2;**	Caudovirales - Siphoviridae
root;Viruses;unclassified bacterial viruses;**Methylophilaceae phage P19250A;**	Caudovirales - Siphoviridae
root;Viruses;unclassified bacterial viruses;**Ralstonia phage DU_RP_I;**	Caudovirales – Podoviridae?
root;Viruses;unclassified bacterial viruses;**Stenotrophomonas phage vB_SmaS-DLP_6;**	Caudovirales - Myoviridae
root;Viruses;unclassified bacterial viruses;**Synechococcus phage S-CAM7;**	Caudovirales - Myoviridae
root;Viruses;unclassified bacterial viruses;**Synechococcus phage S-CAM9;**	Caudovirales - Myoviridae
root;Viruses;unclassified bacterial viruses;**Lactobacillus phage Semele;**	Unclassified bacteriophages
root;Viruses;unclassified bacterial viruses;**Pontimonas phage phiPsal1;**	Unclassified bacteriophages
root;Viruses;unclassified bacterial viruses;**Pseudomonas phage pf16;**	Caudovirales - Myoviridae
root;Viruses;unclassified bacterial viruses;**Paracoccus phage Shpa;**	Caudovirales - Siphoviridae
root;Viruses;unclassified bacterial viruses;**Synechococcus phage S-EIVl;**	Caudovirales - Unclassified
root;Viruses;unclassified bacterial viruses;**Alteromonas phage PB15;**	Caudovirales - Siphoviridae
root;Viruses;unclassified bacterial viruses;**Acidovorax phage ACP17;**	Caudovirales - Myoviridae
root;Viruses;unclassified bacterial viruses;**Streptomyces phage BRock;**	Caudovirales - Myoviridae
root;Viruses;unclassified bacterial viruses;**Mycobacterium phage B1;**	Caudovirales

root;Viruses;unclassified bacterial viruses;**Erwinia phage vB_EamM_Caitlin;**	Caudovirales - Myoviridae
root;Viruses;unclassified bacterial viruses;**Vibrio phage 1.046.O._10N.286.52.E3;**	Unclassified bacteriophages
root;Viruses;unclassified bacterial viruses;**Xanthomonas phage XacN1;**	Caudovirales - Myoviridae
root;Viruses;unclassified bacterial viruses;**Nostoc phage A1;**	Caudovirales - Myoviridae
root;Viruses;unclassified bacterial viruses;**Pseudoalteromonas phage PHS21;**	Unclassified bacteriophages
unclassified dsDNA phages	
root;Viruses;dsDNA viruses, no RNA stage;**unclassified dsDNA phages;**	Unclassified bacteriophages
root;Viruses;dsDNA viruses, no RNA stage;unclassified dsDNA phages;**Idiomarinaceae phage 1N2-2;**	Unclassified bacteriophages
root;Viruses;dsDNA viruses, no RNA stage;unclassified dsDNA phages;**Methylophilales phage HIM624-A;**	Caudovirales – Podoviridae
root;Viruses;dsDNA viruses, no RNA stage;unclassified dsDNA phages;**Salicola phage CGphi29;**	Unclassified bacteriophages
root;Viruses;dsDNA viruses, no RNA stage;unclassified dsDNA phages;**Cyanophage KBS-S-2A;**	Caudovirales - Siphoviridae
root;Viruses;dsDNA viruses, no RNA stage;unclassified dsDNA phages;**Synechococcus phage S-CBP3;**	Caudovirales – Podoviridae
unclassified dsDNA viruses	
root;Viruses;dsDNA viruses, no RNA stage;**unclassified dsDNA viruses;**	Other dsDNA viruses
root;Viruses;dsDNA viruses, no RNA stage;unclassified dsDNA viruses;**Emiliania huxleyi virus PS401;**	Phycodnaviridae

root;Viruses;dsDNA viruses, no RNA stage;unclassified dsDNA viruses;**Phaeocystis globosa virus 14T;**	Mimiviridae
root;Viruses;dsDNA viruses, no RNA stage;unclassified dsDNA viruses;unclassified archaeal dsDNA viruses;Haloviruses;**Halovirus HVTV-1;**	Unclassified bacteriophages
Phycodnaviridae	
root;Viruses;dsDNA viruses, no RNA stage;Phycodnaviridae;environmental samples <Phycodnaviridae>;**Organic Lake phycodnavirus;**	Mimiviridae
root;Viruses;dsDNA viruses, no RNA stage;Phycodnaviridae;environmental samples <Phycodnaviridae>;**Organic Lake phycodnavirus 1;**	Mimiviridae
root;Viruses;dsDNA viruses, no RNA stage;Phycodnaviridae;environmental samples <Phycodnaviridae>;**Organic Lake phycodnavirus 2;**	Mimiviridae
root;Viruses;dsDNA viruses, no RNA stage;Phycodnaviridae;unclassified Phycodnaviridae;**Aureococcus anophagefferens virus;**	Mimiviridae
root;Viruses;dsDNA viruses, no RNA stage;Phycodnaviridae;unclassifiedPhycodnaviridae;**Chrysochromulina ericina virus;**	Mimiviridae
root;Viruses;dsDNA viruses, no RNA stage;Phycodnaviridae;unclassified Phycodnaviridae;**Phaeocystis pouchetii virus;**	Mimiviridae
root;Viruses;dsDNA viruses, no RNA stage;Phycodnaviridae;unclassified Phycodnaviridae;**Pyramimonas orientalis virus;**	Mimiviridae
root;Viruses;dsDNA viruses, no RNA stage;Phycodnaviridae;Prymnesiovirus;unclassified Prymnesiovirus;**Phaeocystis globosa virus [16T];**	Mimiviridae
